# Multiobjective Optimization of Cutting Parameters for TA10 Alloy Deep-Hole Drilling

**DOI:** 10.3390/ma15124366

**Published:** 2022-06-20

**Authors:** Yazhou Feng, Huan Zheng, Xiaolan Han, Zhanfeng Liu

**Affiliations:** Mechanical Engineering College, Xi’an Shiyou University, Xi’an 710065, China; asian5921@126.com (Y.F.); hanxiaolang007@163.com (X.H.); lzhfg9@163.com (Z.L.)

**Keywords:** deep-hole drilling, TA10, chip morphologies, tool wear, hole-axis deflection

## Abstract

In order to obtain better quality TA10 pipes, the Boring and Trepanning Association (BTA) deep-hole drilling process is used. However, this type of machining leads to difficult chip removal, tool wear, and poor hole-surface quality. In this study, a deep-hole drilling experiment was conducted on TA10 workpieces using the designed tool with different process parameters, and the process parameters were optimized by machining results with multiple objectives such as chip morphologies, tool wear, hole-axis deflection, and hole surface roughness. The results show that different process parameters have a great impact on the cutting process, with a higher feed resulting in smoother chip removal and a lower spindle speed resulting in lighter tool wear and less hole axis deflection. When the spindle speed is 145 r/min and the feed is 0.12 mm/r, the machined TA10 pipe meets both the accuracy requirement of roughness and the machining efficiency.

## 1. Introduction

TA10 alloy is a near-alpha-phase titanium alloy developed to improve the crevice corrosion performance of pure titanium and is often used in many fields such as chemical, metallurgical, and petroleum [1,2]. TA10 pipes have good corrosion resistance, cold forming properties, and weldability and are widely used in pipeline environments with complex service environments [3,4]. Although TA10 alloy has excellent mechanical properties, it is a typically difficult-to-machine material; its high strength and high hardness lead to high requirements for deep hole drilling; and it poses a challenge due to the closed environment, the difficulty of observation, and the difficulty of chip removal [5,6]. The deep-hole drilling system for machining TA10 alloy can be divided into gun drilling system, BTA system, jet suction drilling system, and DF system according to its chip removal method and cooling method [7,8]. Among them, the BTA system is widely used due to its advantages of high machining quality, wide machining range, and stable performance [9]. At the same time, the BTA system has a relatively simple structure, easy operation, and higher system rigidity compared with the gun drilling system; it also has better chip removal and cooling lubrication, which is convenient for observing the tool wear condition [10,11]. Therefore, BTA deep-hole drilling is widely used. When processing TA10 workpieces, the cutting force is not large, but the contact area between the tool and the chip is small and the stress is greater, making the processing tool extremely easy to wear; further, the workpiece is very easy to rebound deformation [12,13], seriously affecting the processing surface quality and causing unnecessary economic losses.

To obtain a good quality hole surface and high machining efficiency, machining conditions such as tool geometry parameters, process parameters, and workpiece material properties need to be fully considered [14,15]. Among them, tool geometry parameters include rake angle, relief angle, tool approach angle, drill tip eccentricity and chip breaker size, etc., while process parameters mainly include spindle speed and feed [16]. The selection of reasonable tool geometry and process parameters can make the chip morphologies, tool wear, hole-axis deflection, and hole surface roughness meet the machining requirements, thus improving the machining stability, machining quality, and tool life [17,18,19]. At the same time, the merits of the process parameters are mainly evaluated by the chip morphologies, tool wear, hole-axis deflection, and hole surface roughness. Currently, Process parameters mainly affect chip deformation and chip breakage. Li et al. [20] analyzed the factors affecting chip breakage in staggered-tooth BTA deep-hole drilling using chip formation and chip bending deformation mechanism flowing through the rake face and chip breaker. Tian et al. [21] derived the conditions for complete geometric chip breakage based on an in-depth analysis of the kinematic characteristics of ultrasonic assisted drilling (UAD). Different process parameters lead to different levels of tool wear, which in turn, affect the hole surface roughness [22,23]. Han et al. [24] performed deep-hole boring experiments on pure niobium tubes and used the results of the experiments to gradually adjust the tool geometry and machining process parameters to obtain an optimal set of cutting parameters that minimized tool wear and achieved a surface roughness of 3.2 µm on the inner hole of pure niobium tubes. Abdelhafeez et al. [25] conducted mechanical drilling experiments on CR4 steel using double-edge twist drills of different diameters to investigate the effect of feed and tool diameter on tool rear face wear and drilling quality. Khanna et al. [26] conducted a drilling experiment on Inconel 718 alloy using cryogenic machining and analyzed the torque, thrust, tool wear, chip morphologies, and hole surface roughness generated during machining. Al-Tameemi et al. [27] investigated the effect of process parameters and three types of tool coatings (TiN/TiAlN, TiAlN, and TiN) on the surface finish, shape, and dimensional tolerances of drilled holes. In terms of hole-axis deflection, Berend et al. [28] designed a new measurement system that can measure the hole-axis deflection while drilling and determine the deflection by calculating the bending line of the boring bar using the corresponding algorithm. Gerken et al. [29] developed a compensation unit to compensate the straightness deviation during drilling and investigated the effect of the compensation unit on the straightness deviation by finite element simulation. However, few studies have been mentioned in the literature on chip morphologies, tool wear, hole-axis deflection, and hole surface roughness in deep-hole drilling of TA10 workpieces based on different process parameters.

Therefore, to obtain a set of process parameters with good chip morphologies, tool wear, hole-axis deflection, and hole surface roughness, taking the TA10 workpiece as the research object, the BTA deep-hole drilling experiment is carried out. In Section 2, the procedure of the experiment and the structure of the tool are described. In Section 3, the influence of different combinations of process parameters on the chip morphology, tool wear, hole-axis deflection, and hole surface roughness is analyzed and the optimal parameters for deep-hole drilling of TA10 are obtained.

## 2. Experimental Conditions and Methods

### 2.1. Specimens and Experimental Process

The specimens to be machined in this experiment are TA10 titanium alloy bars; their chemical composition and physical properties are shown in Table 1 and Table 2, respectively. The outer diameter of the specimen is 120 mm and the length is 2700 mm. Four bars were investigated. The inner diameter of the final product is 54 mm and the length is 2700 mm. The roughness required for the final product is 6.3 µm.

**Figure 1:**
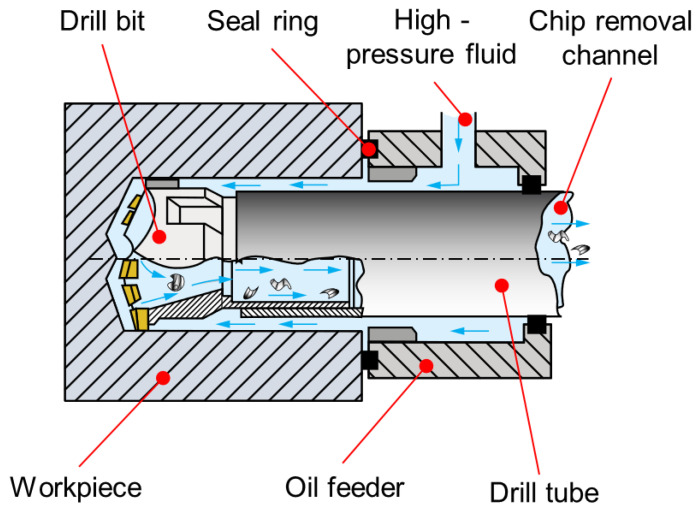
Schematic diagram of the BTA system.

**Figure 2:**
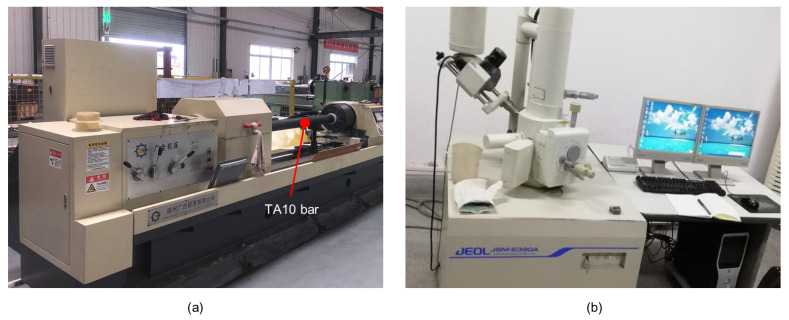
Processing environment and experiment equipment. (**a**) T2120G deep-hole drilling and boring machine (Dezhou, China); (**b**) scanning electron microscope JSM-6390A (Akishima, Japan).

The experiment was carried out on a T2120G deep-hole drilling and boring machine for a BTA system, as shown in Figure 1 and Figure 2, which uses a workpiece rotation and tool feed. As the workpiece is drilled, high-pressure fluid enters through the oil feeder and flows to the cutting area through the annular space between the inner wall of the workpiece and the outer wall of the drill tube. Subsequently, the chips that have broken away from the workpiece are discharged from the chip removal channel by the high-pressure cutting fluid [30,31]. Due to the small diameter of the chip removal channel of the tool, it is difficult for chips to be discharged from the chip removal channel when drilling deep holes of less than ϕ 16 mm. Therefore, the BTA deep-hole drilling system mainly processes holes from ϕ 18∼65 mm [7,8].

To analyze the influence of different process parameters on the machining state, the spindle speed was set to 145 r/min and 195 r/min, and the feed was set to 0.06 mm/r and 0.12 mm/r. To facilitate chip removal and reduce the cutting temperature, HN-69-1 emulsion with a cutting fluid pressure of 3 MPa and a flow rate of 150 L·min${}^{-1}$ was selected in this paper.

Chip morphologies, tool wear, hole-axis deflection, and hole surface roughness are studied in the results of the experiment. The scanning electron microscope (SEM) JSM-6390A and its accompanying energy dispersive spectrometer (EDS) were used to further observe the tool wear of the cutting edge and analyze the chemical elemental composition of its surface. The ultrasonic thickness gauge was used to measure the wall thickness of TA10 pipes and the hole-axis deflection was calculated on the wall thickness. The TR200 handheld roughness meter was used to measure the surface roughness of the inner hole.

### 2.2. Structure of the Tool

**Figure 3:**
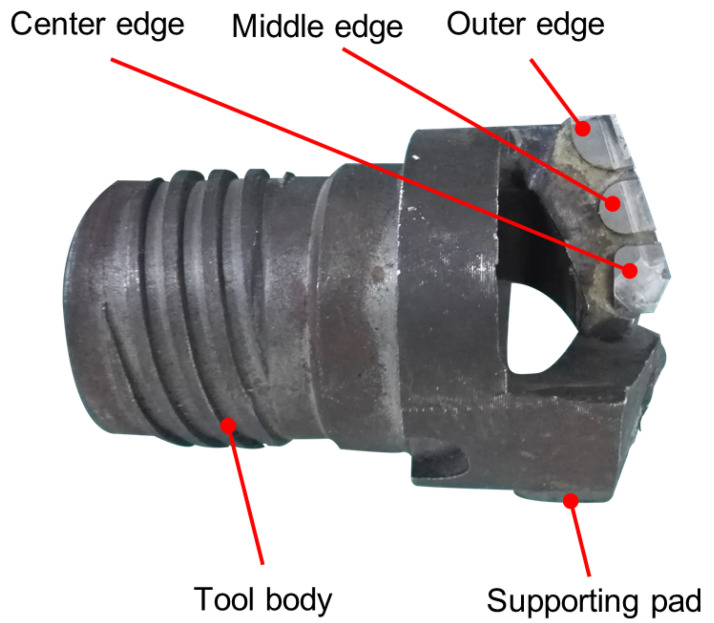
Structure of the cutting tool.

As shown in Figure 3, the staggered teeth BTA drill with five teeth is used in the experiment, which is conducive to chip breakage and chip removal. Although this structure is more complicated than the single-tooth BTA drill, the cutting force and torque are smaller, the chip removal effect is excellent, and the guidance is stable, which can meet the processing requirements. YG8 Carbide has high impact toughness, vibration resistance, and a low affinity for titanium elements; so, YG8 carbide was chosen as the BTA cutting edge material [32,33]. The geometric parameters of the BTA drill have an important influence on deep-hole drilling; the geometric parameters of the BTA drill are selected as follows.

The value of rake angle of the BTA drill depends on the material property and processing requirements [34]. Due to the small plastic deformation and high chemical activity of TA10, the contact length between the rake face and the chip is small, the cutting stress and cutting temperature are relatively concentrated, and the hardened layer is easily produced on the rake face; so, a smaller rake angle should be used to strengthen the edge strength and increase the contact area between the chip and the rake face. Therefore, the rake angle was set to 3°.

The value of relief angle of the BTA drill also depends on the material property. Due to the small elastic modulus of TA10, the rebound under the cutting layer is serious [35]. To reduce the wear of the rear face of the tool and make the cutting edge easy to cut into the metal layer, a larger relief angle helps to improve the durability of the tool, reduce the rebound deformation of the workpiece, and reduce the heat generation; thus, a relief angle of 8° was chosen.

To reduce the radial force of the drill bit, prevent the drill bit from being deflected, and facilitate chip removal, the tool approach angle of the outer cutting edge was set to 18°, tool approach angle of the inner cutting edge to 20°, and the drill tip eccentricity to 4 mm. Since the hole diameter and feed rate are relatively large, the width of the chip-breaker ($W_{n}$) is taken as the larger value of 2 mm, and the depth of the chip-breaker ($H_{n}$) is taken as 0.4 mm.

**Figure 4:**
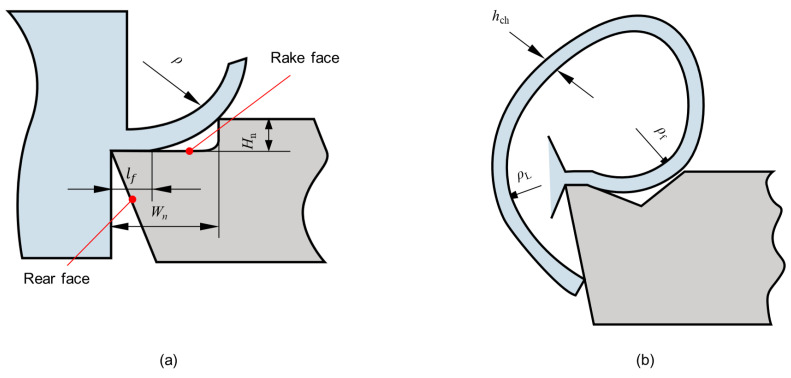
Chip breakage process diagram. (**a**) Chip bending process. (**b**) Chip breakage process.

Chip breakage is the main problem for BTA drilling of the TA10 material; so, the maximum strain theory was used as the chip breakage criterion. Chip breakage occurs when the maximum strain is greater than the fracture strain. Figure 4 shows the chip breakage process. During the chip flow, the chip is subjected to a positive bending strain by the chip breaker. When the chip end touches the rear surface, the chip generates a reverse bending strain, and the chip repeatedly bends and breaks when the bending strain is greater than the material limit strain. Thus, it can be seen that the combined effect of forward bending strain and reverse bending strain causes the chip to break. The chip breaker size affects the chip curl radius (ρ), which can be calculated from the following Equation (1):

(1) $$\rho =\frac{{\left(W_{n}-l_{f}\right)}^{2}}{2H_{n}}+\frac{H_{n}}{2}$$

where $l_{f}$ is chip contact length, $W_{n}$ is the width of the chip breaker, and $H_{n}$ is the height of the chip breaker.

The forward bending strain ($\varepsilon_{wp}$) is expressed by the following Equation (2):

(2) $$\varepsilon_{wp}=\pm \frac{h_{ch}}{2\rho_{f}}$$

where $h_{ch}$ is chip thickness and $\rho_{f}$ is chip radius after chip flow through chip breaker.

The bending strain at reverse fracture ($\varepsilon_{wnb}$) can be expressed as

(3) $$\varepsilon_{wnb}=\frac{h_{ch}}{2}\left(\frac{1}{\rho_{f}}-\frac{1}{\rho_{L}}\right)$$

where $\rho_{L}$ is chip radius at chip reverse fracture.

Therefore, the total bending fracture strain ($\varepsilon_{wb}$) can be expressed as

(4) $$\varepsilon_{wb}=\varepsilon_{wp}+\varepsilon_{wnb}=\frac{h_{ch}}{2}\left(\frac{2}{\rho_{f}}-\frac{1}{\rho_{L}}\right)>\varepsilon_{b}$$

When $\varepsilon_{b}$ is greater than the ultimate strain of the workpiece material, the chip fractures. From Equation (4), it can be seen that when the feed is larger and the chip $h_{ch}$ is larger, the chip is more likely to break; when the chip breaker size is suitable and the curl radius becomes smaller, the chip is also easy to break.

## 3. Results and Discussion

### 3.1. Chip Morphologies and Tool Wear

In deep-hole drilling, the chips are discharged through the narrow channel inside the BTA tool holder under the action of high-pressure cutting fluid, and the smooth discharge of chips is the prerequisite for continuous deep-hole drilling. When the cutting fluid flow rate, pressure, and chip removal space are certain, the chip morphologies determine to a certain extent whether the chip can be discharged smoothly. According to different spindle speeds (*n*) and feeds (*f*), BTA deep-hole drilling experiments were carried out on the TA10 bar. The macro chip morphologies and drilling conditions obtained are shown in Table 3.

**Figure 5:**
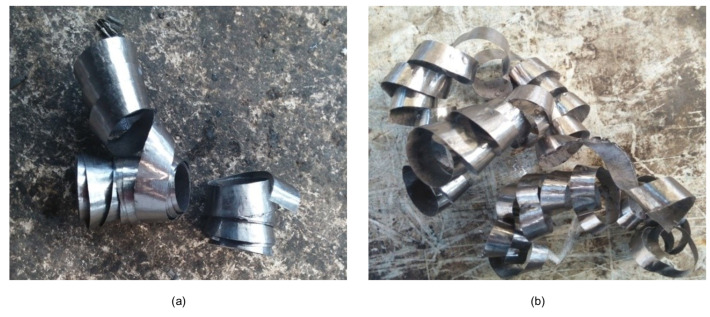
Chip morphologies. (**a**) Short spiral chips. (**b**) Long coiled chips.

The experimental results show that the process parameters directly affect the chip morphologies and tool wear, where the chip morphologies are mainly related to the feed. When the feed is 0.12 mm/r, short spiral chips are produced, as shown in Figure 5a, which is due to $\varepsilon_{wb}$ becoming larger when the $h_{ch}$ increases at a higher feed rate, resulting in easier chip breakage and, thus, the formation of short spiral chips, which facilitates chip removal. However, when the feed is 0.06 mm/r, long coiled chips are generated, as shown in Figure 5b; when the chip length increases significantly, which is due to the smaller feed and smaller $h_{ch}$, the less likely the chip is to break. At the same time, as the drilling depth continues to increase, the thinner chips become entangled due to insufficient cutting fluid pressure.

Therefore, the chips generated by the higher feed can be discharged more smoothly.

Based on the SEM and its attached EDS, the wear of the BTA drill bit is analyzed by the morphological characteristics of the tool surface and the chemical composition covered by the surface, and the SEM and EDS analysis was performed after each TA10 bar was drilled. Since the center edge is in the worst cutting condition among several cutting edges, the cutting force of the cutting teeth is the largest and the extrusion is the most serious; the analysis is mainly carried out on the rake or rear face of the center edge of the BTA drill bit.

**Figure 6:**
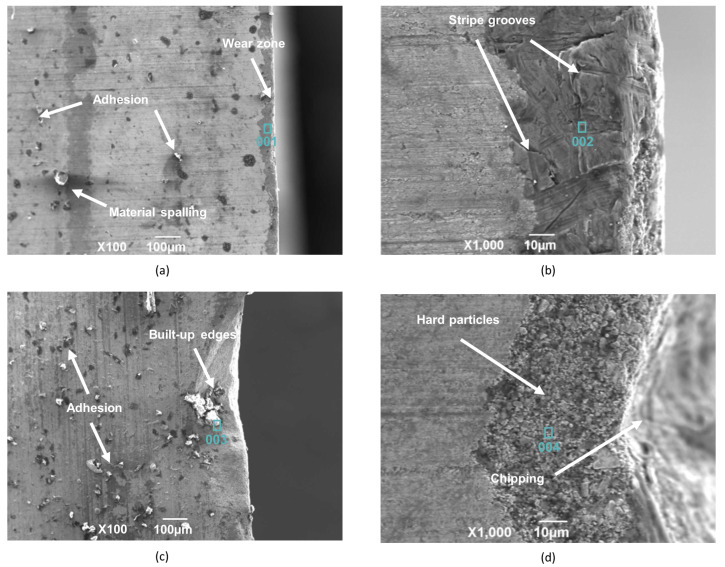
Wear on the center edge of the drill bit during TA10 deep-hole drilling. (**a**) *n* = 145 r/min and *f* = 0.06 mm/r; (**b**) *n* = 145 r/min and *f* = 0.12 mm/r; (**c**) *n* = 195 r/min and *f* = 0.06 mm/r; (**d**) *n* = 195 r/min and *f* = 0.06 mm/r.

**Figure 7:**
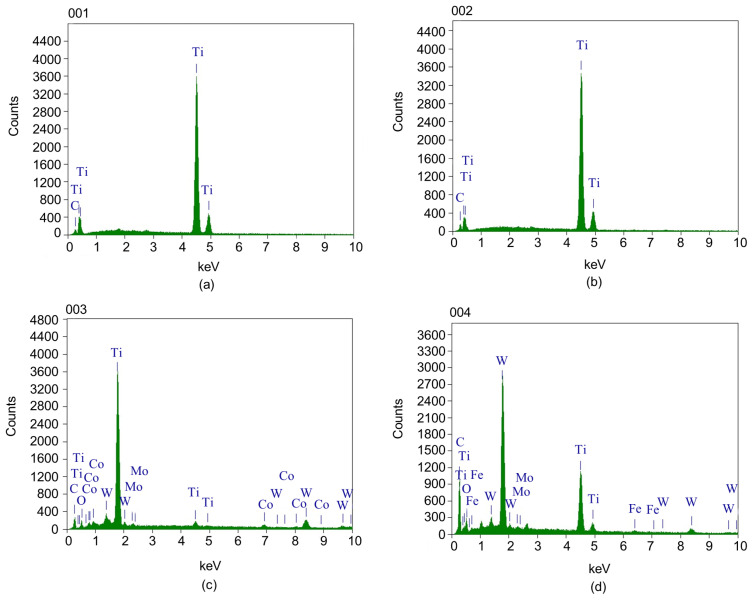
Energy spectrum analysis. (**a**) 001 region; (**b**) 002 region; (**c**) 003 region; (**d**) 004 region.

As shown in Figure 6a, when the spindle speed is 145 r/min and the feed is 0.06 mm/r, a certain amount of material spalling and adhesion wear occurs on the rake face of the center edge. This is caused by the uneven distribution of the tool and workpiece materials. The chips carried away the impurities on the cutting edge and adhesion wear occurred under the high temperature and extrusion conditions. A shallow wear zone appeared at the edge of the cutting edge, and the 001 region was selected for energy spectrum analysis. As shown in Figure 7a, the 001 region is rich in a large amount of Ti and a small amount of C. This indicates that during machining, the workpiece material diffuses to the tool surface under high temperature and pressure and diffusion wear occurs.

When the spindle speed is 145 r/min and the feed is increased to 0.12 mm/r, as shown in Figure 6b, there are many hard spots formed by the workpiece material bonded on the rear face of the center edge, a wear zone is formed at the tool edge, and there are also many stripe grooves on the wear zone. This is because the impurities such as carbides present in the TA10 workpiece material have scratched a groove on the surface of the tool, thus forming hard point wear. To further analyze the wear zone on the rear face of the center edge, the 002 region was selected for energy spectrum analysis. As shown in Figure 7b, it can be found that the elements in the 002 region are more consistent with those in the 001 region, where the diffusion wear and hard point wear occurred.

When the spindle speed is increased to 195 r/min and the feed is 0.06 mm/r, a lot of adhesion is formed by the workpiece material on the rake face of the center edge, as shown in Figure 6c. At the same time, there are obvious built-up edges near the cutting edge, which are formed by the direct contact of the cutting edge with the workpiece, and the large cutting force and cutting heat promote the adsorption of TA10 material on the tool surface as well as mutual diffusion. Due to the repeated generation and spalling of the built-up edges, significant wear was generated at the cutting edge. The energy spectrum analysis of the selected 003 region is shown in Figure 7c, which is mainly enriched with Ti, Mo, W, Co, and a small amount of O. This indicates that this region not only produces adhesion wear and diffusion wear due to the formation of adhesion and built-up edges under high-temperature conditions but also produces chemical wear by oxidation reaction in the air.

When the spindle speed is 195 r/min and the feed is increased to 0.12 mm/r, large chunks of material are removed by adhesion wear and the matrix of the tool was revealed, as shown in Figure 6d. This is due to the higher speed and feed rate, which generate higher temperature and cutting force during the drilling process, making the adhesion wear more serious, thus causing the phenomenon of material peeling off from the tool surface and even chipping at the cutting edge. The energy spectrum analysis of the selected 004 region is shown in Figure 7d, and it can be found that this region is involved in cutting due to the condition of material spalling, which makes the exposed external hard particles and TA10 material diffuse and bond to each other, and then serious vibration occurs.

The comprehensive study found that when deep-hole machining of TA10 was performed under different process parameters, the workpiece material was not hard and affinity was strong, which led to the wear of the tool mainly based on adhesion wear and accompanied by hard point wear, diffusion wear, and chemical wear under different process parameters. The wear is more serious at the spindle speed of 195 r/min and feed of 0.12 mm/r, and even chipping occurs, while the other process parameters are within the acceptable range.

### 3.2. Hole-Axis Deflection

**Figure 8:**
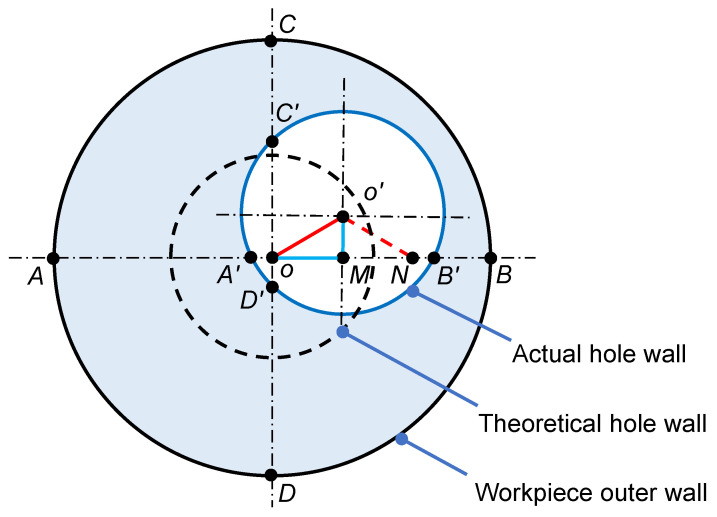
Axial eccentric hole profile.

Hole-axis deflection has a greater impact on the quality of the hole and can be expressed quantitatively by the eccentricity (*e*) of different cross-sections of the machined hole. The *e* was calculated based on the wall thickness value, the wall thickness was measured by ultrasonic thickness gauge at every 300 mm-spacing, and three sets of wall thicknesses were measured at different angles in each cross-section. The *e* of different cross-sections of the machined hole was calculated using Equation (5), and the three eccentricities *e* obtained for each cross-section were summed and averaged. Then, the *e* was integrated and analyzed for the whole workpiece, thus indirectly reflecting the hole-axis deflection of the machined hole. The cross-sectional view of the axial eccentric hole is shown in Figure 8.

(5) $$\begin{array}{ccc} e=OO^{\prime } & =\sqrt{OM^{2}+O^{\prime }M^{2}}\; & =\sqrt{{\left(\frac{AA^{\prime }-BB^{\prime }}{2}\right)}^{2}+{\left(\frac{DD^{\prime }-CC^{\prime }}{2}\right)}^{2}} \end{array}$$

**Figure 9:**
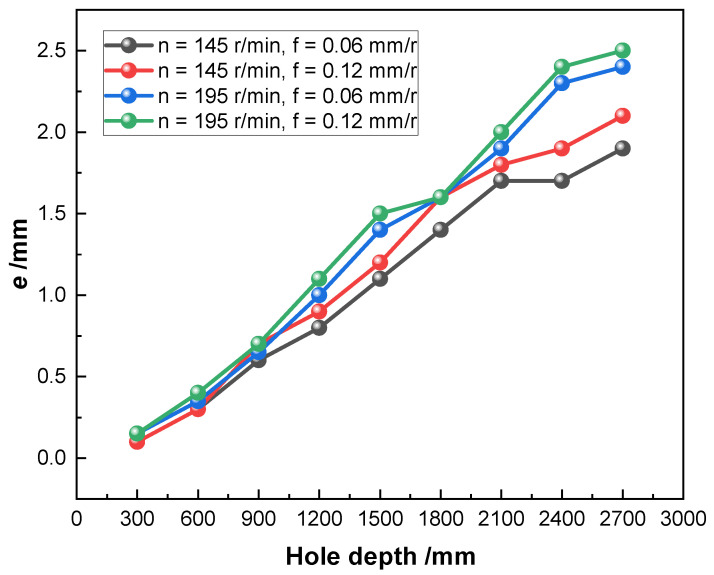
Hole-axis deflection.

From Figure 9, the *e* in this figure is the average of the three eccentricities *e* for each cross-section. It can be seen that *e* increases approximately linearly with the increase in drilling depth, which is since the stiffness of the drill tube gradually decreases with the increase in drilling depth and its vibration becomes more violent, which leads to the gradual increase of *e*.

When the spindle speed is 145 r/min and the feed increases from 0.06 mm/r to 0.12 mm/r, it can be found that the change of the difference between the minimum and maximum values of *e* is relatively small. However, when the spindle speed is increased to 195 r/min, the difference between the minimum and maximum values of *e* of the drilled hole changes greatly. This is due to the low thermal conductivity of the TA10 and the high spindle speed, which leads to difficulty in heat dissipation and increased cutting heat, resulting in increased wear, which in turn, causes vibration in the overall system and a significant variation in the hole-axis deflection of the drilled hole. Another reason for hole-axis deflection is that the drill tip does not coincide with the workpiece’s circle center due to the larger spindle speed in the initial drilling stage, resulting in a large response when drilling; then, a deflection occurs. Therefore, under the condition of large spindle speed, it should be drilled at a low spindle speed in the initial drilling stage and then adjusted to the required spindle speed.

It can be found that the hole-axis deflection is small when spindle speed and feed are small. The spindle speed has a greater effect on the hole-axis deflection compared to the feed. When the spindle speed is 145 r/min and the feed rate is 0.12 mm/r, hole-axis deflection is small and the cutting efficiency can be satisfied with the production requirements.

### 3.3. Hole Surface Roughness

To evaluate the quality of the hole under different process parameters, this experiment uses a TR200 handheld roughness meter to measure and analyze the roughness of the machined hole. The surface roughness is measured at the end of the hole due to the long cantilevered amount of the drill tube, which reduces rigidity and creates a certain amount of vibration at the end of the hole processing.

**Figure 10:**
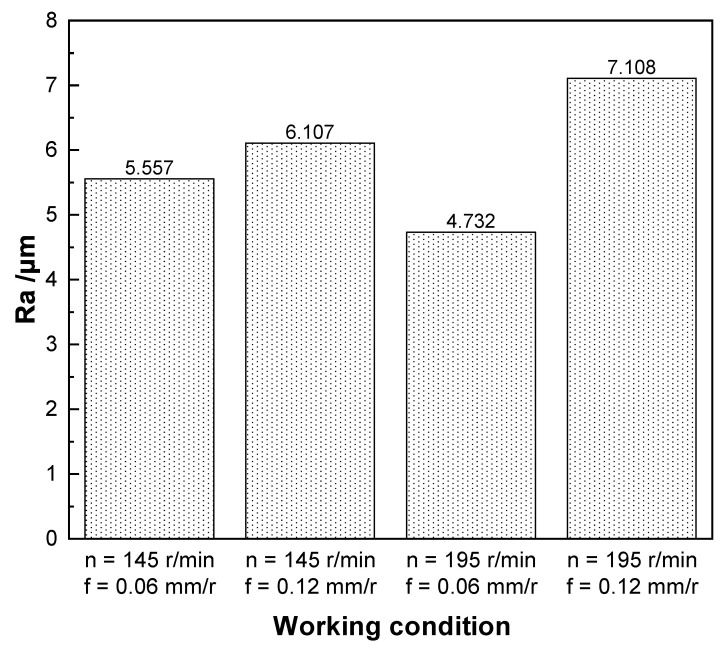
Hole surface roughness.

As shown in Figure 10, it can be found that the arithmetic mean roughness (Ra) is lower when the feed is smaller. Within a certain range, the higher the spindle speed and the smaller the feed, the smaller the roughness. When the spindle speed is 195 r/min and the feed is 0.06 mm/r, the surface roughness is smaller and surface roughness is 4.732 µm, and the quality of the inner hole wall machining is higher. However, the higher cutting speed led to poor chip removal and high cutting temperature, which aggravated the tool wear. Especially when the feed is increased to 0.12 mm/r, the higher speed and feed generate higher temperature and cutting force during the machining process, which increases the tool wear and leads to an increase in the roughness of the machined hole, making the surface roughness greater than 6.3 µm, which exceeds the accuracy requirement. When the spindle speed is 145 r/min, the roughness is more similar in both process parameters, and the surface roughness is less than 6.3 µm. The surface roughness is smaller with a feed of 0.06 mm/r, but the smaller feed will lead to low machining efficiency; so, it cannot meet the machining requirements. When the spindle speed is 145 r/min and the feed is 0.12 mm/r, it meets both the machining accuracy and the machining efficiency.

Therefore, through a comprehensive comparison, combining the chip morphologies, the tool wear, hole-axis deflection, and the surface roughness of the inner hole of TA10 material, it is known that the machining quality and efficiency are better when the spindle speed is 145 r/min and the feed is 0.12 mm/r. Under this process parameter, the short spiral chips produced can be discharged from the drill pipe more smoothly. Although wear is also generated, it is still usable. The hole-axis deflection and hole surface roughness are also relatively small compared with those of other process parameters. For other process parameters, the four objectives had more or less some defects. For example, at a spindle speed of 145 r/min and a feed of 0.06 mm/r, long coiled chips were generated, making chip removal difficult. Although the tool wear, hole-axis deflection, and hole surface roughness are small at this process parameter, the small feed makes machining efficiency low, which greatly increases the economic cost. At the spindle speed of 195 r/min, the wear is more severe and the hole-axis deflection is larger.

## 4. Conclusions

Based on the BTA deep-hole drilling system, a deep-hole drilling experiment was conducted for four TA10 workpieces with geometry ϕ 120 mm × 2700 mm, and the process parameters at different spindle speeds and feeds were used to analyze the impact and effect on multiple objectives such as chip morphologies, tool wear, hole-axis deflection, and hole surface roughness in TA10 deep hole drilling. The following conclusions are drawn.

(1) The process parameters directly affect the chip morphologies, and the chip morphologies are mainly related to the feed; the higher feed generated by the chip can be more smoothly discharged.

(2) The wear of tool is mainly adhesion wear, accompanied by hard point wear, diffusion wear, and chemical wear under different process parameters. At the same time, the wear is more serious at the spindle speed of 195 r/min and feed of 0.12 mm/r, and even chipping occurs, while the other process parameters are within the acceptable range.

(3) The overall hole-axis deflection is smaller when both the spindle speed and feed are smaller, and vice versa. At the same time, the spindle speed has a greater influence on the deflection than the feed.

(4) When the spindle speed is larger and the feed is smaller, the surface roughness is the smallest. However, the higher spindle speed will lead to high cutting temperature, which in turn, leads to serious tool wear; thus, a small feed will not meet the machining efficiency.

(5) In the TA10 alloy deep-hole drilling, in the case of a given geometry of the tool, when the spindle speed is 145 r/min, the feed is 0.12 mm/r, both to meet the requirements of machining accuracy and to meet the processing efficiency.

## Figures and Tables

**Table 1:** Chemical composition of the TA10 (wt%).

Ti	Mo	Ni	Fe	C	N	H	O
99	0.2–0.4	<0.6–0.9	<0.30	<0.08	<0.03	<0.015	<0.25

**Table 2:** Mechanical properties of the TA10.

Tensile Strength (MPa)	Yield Strength (MPa)	Shrinkage (%)	Elongation (%)	Elastic Modulus (GPa)	Density (g·cm${}^{-3}$)	Thermal Conductivity (W·m${}^{-1}$·K${}^{-1}$)	Hardness (HB)
485	345	25	18	112	4.54	19	260

**Table 3:** Machining results under different process parameters.

Number	*n*/(r/min)	*f*/(mm/r)	Chip Morphologies	Cutting Conditions
1	145	0.06	Long coiled chips	Slight vibration and tool wear
2	145	0.12	Short spiral chips	Vibration and tool wear
3	195	0.06	Long coiled chips	Slight vibration and severe tool wear
4	195	0.12	Short spiral chips	Severe vibration and severe tool wear

## Data Availability

Not applicable.

## References

[1] Yang P., Wei Z., Gu X., Cui F., Mao W. (2019). Influences of cold rolling, recrystallization and surface effect on the transformation textures in a TA10 titanium alloy. J. Phys. Conf. Ser..

[2] Yang Y., Wang B., Li Y., Su B., Luo L., Wang L., Huang H., Su Y., Guo J. (2022). Enhanced strength and corrosion resistance in as-cast TA10 alloys via interstitial carbon solute. Mater. Res. Express.

[3] Tang X., Wang S., Qian L., Li Y., Lin Z., Xu D., Zhang Y. (2015). Corrosion behavior of nickel base alloys, stainless steel and titanium alloy in supercritical water containing chloride, phosphate and oxygen. Chem. Eng. Res. Des..

[4] Li J.N., Liu Z.Y., Liu Q., Tian Y. (2019). Microstructure and Oxidation Resistance of Laser-induced Stellite Base Composites on a TA10 Alloy. Lasers in Eng..

[5] Baumann A., Oezkaya E., Schnabel D., Biermann D., Eberhard P. (2021). Cutting-fluid flow with chip evacuation during deep-hole drilling with twist drills. Eur. J. Mech. B/Fluids.

[6] Felinks N., Rinschede T., Biermann D., Stangier D., Tillmann W., Fuß M., Abrahams H. (2022). Investigation into deep hole drilling of austenitic steel with advanced tool solutions. Int. J. Adv. Manuf. Technol..

[7] Biermann D., Bleicher F., Heisel U., Klocke F., Möhring H.C., Shih A. (2018). Deep hole drilling. CIRP Ann..

[8] Chandar J.B., Nagarajan L., Kumar M.S. (2021). Recent Research Progress in Deep Hole Drilling Process: A Review. Surf. Rev. Lett..

[9] Strodick S., Berteld K., Schmidt R., Biermann D., Zabel A., Walther F. (2020). Influence of cutting parameters on the formation of white etching layers in BTA deep hole drilling. tm-Tech. Mess..

[10] Yang S., Tong X., Ma X., Ji W., Liu X., Zhang Y. (2018). The guide block structure design of boring and trepanning association (BTA) deep hole drilling. Int. J. Adv. Manuf. Technol..

[11] Kumar M.S., Deivanathan R. (2021). Effect of process parameters on drilling—An overview. Mater. Today Proc..

[12] Senthilkumar N., Tamizharasan T., Anandakrishnan V. (2014). Experimental investigation and performance analysis of cemented carbide inserts of different geometries using Taguchi based grey relational analysis. Measurement.

[13] Li Y., Kong J., Du D. (2022). Research on deformation mechanism and law of thin-walled flat parts in vacuum clamping. Int. J. Adv. Manuf. Technol..

[14] Singaravel B., Saikrupa C., Sandeep M. (2020). Analysis of Quality Parameters in Drilling of Titanium Alloy. Int. J. Veh. Struct. Syst..

[15] Yuan C.G., Pramanik A., Basak A., Prakash C., Shankar S. (2021). Drilling of titanium alloy (Ti6Al4V)—A review. Mach. Sci. Technol..

[16] Prasanna J., Karunamoorthy L., Raman M.V., Prashanth S., Chordia D.R. (2014). Optimization of process parameters of small hole dry drilling in Ti–6Al–4V using Taguchi and grey relational analysis. Measurement.

[17] Li A., Zhao J., Zhou Y., Chen X., Wang D. (2012). Experimental investigation on chip morphologies in high-speed dry milling of titanium alloy Ti-6Al-4V. Int. J. Adv. Manuf. Technol..

[18] Das S.R., Panda A., Dhupal D. (2017). Experimental investigation of surface roughness, flank wear, chip morphology and cost estimation during machining of hardened AISI 4340 steel with coated carbide insert. Mech. Adv. Mater. Mod. Process..

[19] Liu Z., Liu Y., Han X., Zheng W. (2018). Study on super-long deep-hole drilling of titanium alloy. J. Appl. Biomater. Funct. Mater..

[20] Li X.B., Zheng J.M., Li Y., Kong L.F., Shi W.C., Guo B. (2019). Investigation of chip deformation and breaking with a staggered teeth BTA tool in deep hole drilling. Metals.

[21] Tian Y., Zou P., Yang X., Kang D. (2020). Study on chip morphology and surface roughness in ultrasonically assisted drilling of 304 stainless steel. Int. J. Adv. Manuf. Technol..

[22] Sivaiah P., Bodicherla U. (2020). Effect of surface texture tools and minimum quantity lubrication (MQL) on tool wear and surface roughness in CNC turning of AISI 52100 steel. J. Inst. Eng. (India) Ser. C.

[23] Khanna N., Agrawal C., Dogra M., Pruncu C.I. (2020). Evaluation of tool wear, energy consumption, and surface roughness during turning of inconel 718 using sustainable machining technique. J. Mater. Res. Technol..

[24] Han X., Liu Z., Wang T. (2019). Investigation of tool wear in pull boring of pure niobium tubes. J. Braz. Soc. Mech. Sci. Eng..

[25] Abdelhafeez Hassan A., Li M.J., Mahmoud S. (2020). On miniature hole quality and tool Wear when mechanical Drilling of Mild Steel. Arab. J. Sci. Eng..

[26] Khanna N., Agrawal C., Gupta M.K., Song Q. (2020). Tool wear and hole quality evaluation in cryogenic Drilling of Inconel 718 superalloy. Tribol. Int..

[27] Al-Tameemi H.A., Al-Dulaimi T., Awe M.O., Sharma S., Pimenov D.Y., Koklu U., Giasin K. (2021). Evaluation of cutting-tool coating on the surface roughness and hole dimensional tolerances during drilling of Al6061-T651 alloy. Materials.

[28] Denkena B., Bergmann B., Kaiser S., Mücke M., Bolle D. (2018). Process-parallel center deviation measurement of a BTA deep-hole drilling tool. Procedia Manuf..

[29] Gerken J., Klages N., Biermann D., Denkena B. (2020). In-process compensation of straightness deviation in BTA deep hole drilling using experimental and simulative analysis. Procedia CIRP.

[30] Matsuzaki K., Ryu T., Sueoka A., Tsukamoto K. (2015). Theoretical and experimental study on rifling mark generating phenomena in BTA deep hole drilling process (generating mechanism and countermeasure). Int. J. Mach. Tools Manuf..

[31] Schmidt R., Strodick S., Walther F., Biermann D., Zabel A. (2020). Analysis of the functional properties in the bore sub-surface zone during BTA deep-hole drilling. Procedia CIRP.

[32] Zhu Z., Sui S., Sun J., Li J., Li Y. (2017). Investigation on performance characteristics in drilling of Ti6Al4V alloy. Int. J. Adv. Manuf. Technol..

[33] Wei L., Yang Y., Yang G. (2020). Microstructure and properties of YG8 cemented carbide with different pulse currents. Rare Met..

[34] Li X., Zheng J., Li Y., Kong L., Shi W., Guo B. (2018). Modeling and distribution laws of drilling force for staggered teeth BTA deep hole drill. Math. Probl. Eng..

[35] Volz M., Abele E., Weigold M. (2020). Lateral Vibrations in Deep Hole Drilling Due to Land Width Variation. J. Manuf. Mater. Process..

